# Identification of Peripheral Blood Endotypes Associated With Age in Pediatric Type 1 Diabetes

**DOI:** 10.1155/pedi/5512196

**Published:** 2025-09-27

**Authors:** Laia Gomez-Muñoz, David Perna-Barrull, Paula Sol Ventura, Aina Valls, Francesca Castiello, Marta Vives-Pi, Marta Murillo-Vallés

**Affiliations:** ^1^Immunology Department, Germans Trias i Pujol Research Institute (IGTP), Autonomous University of Barcelona, Badalona 08916, Spain; ^2^Pediatrics Department, Germans Trias i Pujol University Hospital (HGTiP), Autonomous University of Barcelona, Badalona 08916, Spain

**Keywords:** β-cells, age, biomarkers, endotypes, peripheral immune cells, type 1 diabetes

## Abstract

**Aims:** This study aimed to identify age-related peripheral immune endotypes in pediatric patients with type 1 diabetes (T1D) at disease onset and assess their metabolic control 1 year post-diagnosis.

**Methods:** Immune cell subpopulations (T and B lymphocytes, myeloid cells, and natural killer [NK] cells) were analyzed via multicolor flow cytometry in pediatric T1D patients and age- and sex-matched controls, grouped as <7 years, 7–12 years, and >12 years. Sociodemographic, clinical, and metabolic data were collected, including autoantibodies, bicarbonate (HCO_3_), C-peptide, HbA1c, and time in range (TIR), with follow-up for 1 year to evaluate partial remission (PR) likelihood and metabolic control.

**Results:** Patients <7 years showed reduced regulatory immune cells (memory/activated regulatory T lymphocytes (Tregs), regulatory B cells, and Th17) and more severe disease onset (shorter symptoms, greater acidosis, and lower C-peptide). Ages 7–12 exhibited increased memory B cells, particularly the unswitched ones. Myeloid cells showed no significant variation in T1D, despite age trends in controls. Anti-insulinoma-antigen 2 (IA2) titers were lower in patients >12 years, while anti-glutamic acid decarboxylase 65 (GAD65) positivity remained constant. Younger patients had lower PR rates and poorer glycemic control at 1 year.

**Conclusions:** Younger patients face greater immune dysregulation and β-cell loss, while older patients show better immune maturity and metabolic outcomes. These differences underline the need for age-specific T1D therapies.

## 1. Introduction

Type 1 diabetes (T1D) is a chronic autoimmune disorder resulting from the interaction of genetic predisposition and environmental factors. This process leads to the destruction of the insulin-producing β-cells in the pancreas. The molecular mechanisms underlying the autoimmune destruction in T1D remain poorly understood, as does the heterogeneity in its pathogenesis across patients. The progression of T1D is generally described in stages 1–3, with a proposed stage 0 occurring prior to seroconversion and related to genetic risk [1]. However, significant variability in these stages as well as in terms of clinical presentation features exists among individuals, supporting the concept of disease subtypes, which may reflect underlying differences in the pathogenesis and clinical progression. Identifying these distinct features to define specific endotypes is crucial for advancing precision medicine approaches in T1D treatment [2].

Different factors can be used to define these diabetic subtypes, such as age of onset, immune regulation, rate of β-cell destruction, presence of autoantibodies, body weight, genetic predisposition, and environmental exposures. It is well established that age is a key factor influencing the rate of progression during preclinical phases [3]. There is an inverse relationship between age and the likelihood of developing T1D in individuals with autoantibodies, and younger age and earlier seroconversion are associated with a more intense and aggressive autoimmune process [4]. Supporting this, in The Environmental Determinants of Diabetes in the Young (TEDDY) study, it was determined that younger individuals tend to present insulin autoantibodies (IAAs) first, related to DR4–DQ8 haplotype, thus being more prone to develop multiple autoantibodies and to experience an accelerated β-cell loss [5].

Consistent with the more rapid β-cell loss observed in early-onset cases, differences in insulitis have been identified based on age at onset, suggesting the existence of two distinct endotypes. T1D endotype 1 (T1DE1) includes patients diagnosed in early childhood (<7 years) who are characterized by an extensive and early β-cell destruction, an insulitis enriched in CD8^+^ T cells and CD20^+^ B cells, and aberrant processing of insulin [6]. On the other hand, T1D endotype 2 (T1DE2) includes patients diagnosed in adolescence or adulthood (≥13 years) who are characterized by a higher number of insulin-containing islets, normal processing of insulin, and fewer infiltrating CD8^+^ T cells and CD20^+^ B cells [7, 8]. Individuals diagnosed between 7 and 12 years can be defined as either T1DE1 or T1DE2. However, these histopathological differences should be confirmed with a greater number of samples.

Importantly, pediatric individuals diagnosed at a later age tend to exhibit better blood glucose control and experience fewer complications, consistent with pancreatic studies showing greater preservation of β-cells [9]. Also, they are more likely to achieve partial remission (PR) than individuals diagnosed before the age of 5 [10]. Notably, evidence suggests that insulin-containing islets may actively defend themselves against the autoimmune reaction by upregulating nonclassical class I HLA molecules like HLA-F, as well as inhibitory molecules like PD-L1 [11, 12]. These protective mechanisms may be more frequent in patients with the T1DE2.

Currently, the characterization of endotypes is advancing through integrated assessments that combine genetic, immunological, and metabolic analyses [13–15]. These results emphasize that it will be critical to stratify patients appropriately according to their insulitis and immune profile and age at diagnosis (endotypes) to design effective therapies for T1D. In the context of preventive immunotherapy, emerging evidence suggests that T1DE1 may respond more effectively to interventions targeting specific immune cell subsets, such as rituximab or teplizumab [16, 17]. In contrast, glutamic acid decarboxylase (GAD)–alum therapy demonstrates potential efficacy in treating T1DE2 [18].

However, due to the challenges of profiling insulitis in individuals living with T1D, noninvasive techniques capable of identifying specific immunological profiles for each endotype should be incorporated. Therefore, this study aimed to classify a cohort of newly diagnosed T1D patients into three age groups (<7, 7–12, and >12 years) and assess changes in different whole blood immune cell subpopulations and demographic, clinical, and metabolic parameters that could contribute to define peripheral blood endotypes. Additionally, a 1-year follow-up was conducted to further investigate the heterogeneity of the disease.

## 2. Materials and Methods

### 2.1. Study Population

For the characterization of T1D endotypes at diagnosis, 67 pediatric patients with new-onset T1D diagnosed between 1 June 2016 and 30 April 2023 and 38 age- and sex-matched nondiabetic control subjects were included.

Control children were recruited at our hospital during routine outpatient evaluations or pre-surgical procedures for noninflammatory and unrelated conditions. These children had no evidence of acute or chronic inflammatory diseases, nor conditions known to affect immune function and normal body mass index (BMI).

All patients fulfilled the American Diabetes Association classification criteria for T1D, with at least one positive anti-islet autoantibody at disease onset detected by ELISA (to insulinoma-antigen 2 [IA2] or GAD65).

Inclusion criteria for both patients with T1D and control subjects were being under 18 years of age and presenting normal BMI according to the Spanish BMI pediatric cohort growth chart. Exclusion criteria for both patients with T1D and control subjects were being under immunosuppressive or anti-inflammatory treatment, type 2 diabetes, pregnancy, and receiving drugs that could alter glycemia.

### 2.2. Sample Collection, Clinical and Demographic Determinations, and Longitudinal T1D Follow-up

At disease onset, all samples were collected between 1 and 14 days after diagnosis. Blood samples of 6 mL were obtained from newly diagnosed patients with T1D and nondiabetic control subjects in EDTA tubes (BD Biosciences, San Jose, CA, USA) and processed within 6 h. Patients were followed for 12 months.

Demographic descriptors on each patient were collected, including age, sex and first-grade relatives affected with T1D. Age at diagnosis was used to stratify the data and to explore differences between the different age groups: <7 years, 7–12 years, and >12 years. Clinical characteristics at diagnosis included BMI SD score (SDS) based on the Spanish BMI pediatric cohort growth chart data, duration of previous symptoms related to diabetes, and the presence of other autoimmune diseases (e.g., celiac disease or thyroiditis).

Blood samples from patients with T1D at diagnosis were acquired for centralized measurement of HbA1c by high-performance liquid chromatography (ADAMS A1c HA-8180V, Arkray, MN, USA), base excess (BE) and plasma bicarbonate (HCO_3_) to assess the degree of ketoacidosis, and fasting basal C-peptide (ng/mL) by chemiluminescence immunoassay (Architect i2000, Abbott, IL, USA) to determine pancreatic reserve. Insulin requirements were also recorded.

At 12 months follow-up, metabolic control was evaluated by HbA1c, fasting basal C-peptide and time in range (TIR), value obtained from continuous glucose monitoring data for the last 14 days.

### 2.3. Detection of PR in T1D Patients

To assess PR, we calculated the insulin dose-adjusted HbA1c (IDAA1c) using both HbA1c levels and insulin requirements, following the formula: HbA1c (%) + (4 × insulin dose [U/kg/day]). An IDAA1c of nine or lower indicated the PR phase [19]. Since the highest proportion of patients in PR is observed within 2–6 months postdiagnosis, those not meeting PR criteria after 8 months were classified as nonremitters.

### 2.4. Extended Immunophenotyping

A phenotypic analysis of immune subpopulations—including distinct subsets of T lymphocytes, B lymphocytes, monocytes, natural killer (NK) cells, and dendritic cells (DCs)—was conducted in T1D patients at diagnosis (*n* = 67) and in nondiabetic control subjects (*n* = 38). Fresh whole blood samples of 1–2 mL were washed with 15 mL of FACSFlow Sheath Fluid (ThermoFisher Scientific, Waltham, MA, USA), followed by staining 100 μL of blood with specific monoclonal antibodies for 20 min at room temperature, shielded from light. Antibody panels were constructed as previously described [20, 21]. After incubation, erythrocytes were lysed for 7 min using Lysing Buffer (BD Biosciences), and samples were then washed and resuspended in FACSFlow Sheath Fluid. A minimum of 10,000 events per sample were acquired with a 3-laser FACS Canto II and a 4-laser LSR Fortessa Flow Cytometers (BD Biosciences) and analyzed using FACSDiva software (BD Biosciences). Necrotic and apoptotic cells were excluded from the analysis based on their FSC-A/SSC-A properties and doublets were excluded by FSC-A/FSC-H. The gating strategy to analyze specific leukocyte subsets was based on international consensus [22] and as shown in [20]. Fluorescence minus one controls were used to define CCR7 expression on Th17 lymphocytes and PTK7 expression on recent thymic emigrants. Furthermore, internal reference populations were used as positivity controls in the analysis of CCR7 vs. CD45RA in panel 1, CD27 vs. CD24 in panel 2, CD19 vs. CD21 in panel 3, CCR4 vs. CCR7 in panel 4, and CD45RO vs. CCR4 plus CD45RO vs. HLA-DR in panel 5.

### 2.5. Ethics Statement

All the experiments were carried out in strict accordance with the principles outlined in the Declaration of Helsinki for human research and after the approval of the Committee on the Ethics of Research of the Germans Trias i Pujol University Hospital (protocol code PI-19-010 approved on 25 January 2019 and PI-23-128 approved on 18 October 2023). Informed consent was obtained from all subjects/legal representatives involved in the study.

### 2.6. Statistical Analysis

Differences between two groups of unpaired data were analyzed using the nonparametric two-tailed Mann–Whitney test. Fisher's exact test was used for the analysis of categorical variables. All analyses were performed with GraphPad Prism 9.0 (GraphPad Software Inc, San Diego, CA, USA) and R 4.3.2. A *p*-value of ≤0.05 was considered statistically significant.

## 3. Results

### 3.1. Pediatric Patients Under the Age of 7 Have More Severe T1D at Diagnosis

Clinical features and metabolic data from patients with T1D divided by age at diagnosis (<7, 7–12, and >12) are summarized in Table 1. In total, 67 patients (56.7% men) with an average age of 9.7 years were recruited in the study. 21 patients (31.3%) belong to group <7 years, 26 (38.8%) to the group 7–12 years and 20 (29.8%) to the group >12 years. No statistically significant differences were found in BMI SDS or sex between age groups, although male sex predominates in the <7 years group (71.4% vs. 42.3% and 60%). No differences were found in the percentage of relatives with T1D, the percentage of patients with other autoimmune diseases, or insulin doses. Patients <7 years had symptoms for less time (mean 2.2 weeks) than those older than 7 or 12 years (5.3 and 6.4 weeks). In addition, HCO_3_ and BE levels were significantly lower in patients <7 years compared to those older than 12 years, indicating greater metabolic acidosis at diagnosis. As for the percentage of HbA1c, levels were lower in <7 years (mean 10.3% [9.4–10.6]) and increased progressively with age (11.9% [10.7–13.1] and 12.4% [10.7–14.3]). However, younger patients also had lower C-peptide levels (mean 0.27 ng/mL [0.1–0.3]), increasing from the age of 7 years and progressively with age (0.39 ng/mL [0.2–0.5] and 0.56 ng/mL [0.3–0.6]). Regarding anti-IA2 and anti-GAD65 autoantibodies, we could observe that there is a lower proportion of patients positive for IA2A when diagnosed at age >12 years, presenting significantly lower autoantibody titers compared to the 7–12 years age group. By contrast, although a higher proportion of individuals tested positive for GAD65A in all groups, no significant differences were observed when comparing the titers or the percentage of positives. To sum up, younger patients (<7 years) have more severe disease at diagnosis, with more metabolic acidosis and lower C-peptide. Immunological differences are more evident in IA2A titers between >12 years and the other groups, with older patients having lower titers. These differences suggest variations in the pathophysiology and clinical presentation of T1D according to age.

### 3.2. Pediatric Patients Over the Age of 12 Show Better Glycaemic Control and Higher Residual β-Cell Function at 12 Months Post-Diagnosis

Next, in Table 2, we analyzed metabolic control 1 year after diagnosis to compare between age groups (<7, 7–12, and >12 years). At 12 months after diagnosis, C-peptide levels were significantly lower in the <7 years group (mean 0.1 ng/mL [0.01–0.1]) compared to the older age groups (0.46 ng/mL [0.2–0.65] for the 7–12 years group and 0.95 ng/mL [0.4–1.5] for the >12 years group). Significant differences were also observed between the 7–12 years and >12 years groups, so that β-cell function is better preserved in older patients at diagnosis. HbA1c levels were similar between the <7 years (mean 7.6% [7–8.1]) and 7–12 years (7.6% [7.1–8.5]) groups, but significantly lower in the >12 years group (6.7% [5.9–7.2]). Thus, the >12 years group achieves better glycaemic control at 12 months compared to the younger groups. Finally, the TIR, which measures the percentage of time that blood glucose levels are in the target range, was higher in the >12 years group (mean 67.4% [53–75]) compared to patients <7 years (51.7% [41.5–63]) or between 7 and 12 years (47.8% [30–60]), suggesting that older patients can maintain blood glucose levels in the target range for longer. Ultimately, these findings highlight important differences in the progression and management of T1D according to age at diagnosis.

### 3.3. Pediatric Patients Over the Age of 7 Experience PR More Frequently Than Younger Patients

After stratifying T1D patients by age groups and assessing remission status based on an IDAA1c <9, we observed that 52.4% of patients under 7 years of age experienced remission (11 out of 21 patients). Similarly, 65.4% of patients between 7 and 12 years old were also in PR after 2–6 months (17 out of 26 patients). However, patients diagnosed at 13 years or older had a higher likelihood of achieving remission, with 85% meeting remission criteria (17 out of 20 patients; Table 3). Therefore, the age at diagnosis is a key factor influencing the likelihood of experiencing PR. This may be closely related to the fact that older patients tend to have a greater pancreatic reserve, with significantly higher fasting baseline C-peptide levels than younger patients (Table 1). Moreover, younger patients at diagnosis presented lower levels of HbA1c in comparison with the intermediate group or the older one, indicating a faster transition to the symptomatic stage 3 in the youngest (Table 1).

### 3.4. Younger Pediatric Patients With T1D Course With Decreased Levels of Regulatory T and B Lymphocytes and Memory B Cells

**Figure 1:**
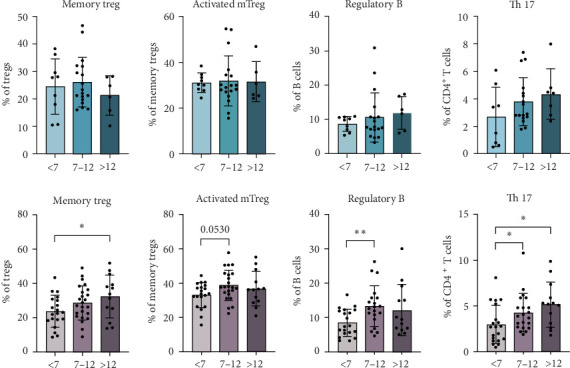
Age-related differences in regulatory T and B lymphocytes at diagnosis of T1D. The percentages of memory and activated Tregs, Bregs, and Th17 were determined in nondiabetic control subjects (blue bars) or at the time of T1D diagnosis (pink bars) and compared between age groups (<7, 7–12, and >12). *⁣*^*∗*^*p*  < 0.05, *⁣*^*∗∗*^*p*  < 0.01, nonparametric *t*-test.

The analysis of immune cell subpopulations revealed changes across the different age groups in pediatric T1D that were not present in control nondiabetic subjects also separated by age groups. Younger patients with T1D (<7 years) have diminished percentages of memory regulatory T lymphocytes (Tregs), activated Tregs, regulatory B lymphocytes (Bregs), and Th17 cells, especially when compared with the intermediate group (7–12 years) (Figure 1).

**Figure 2:**
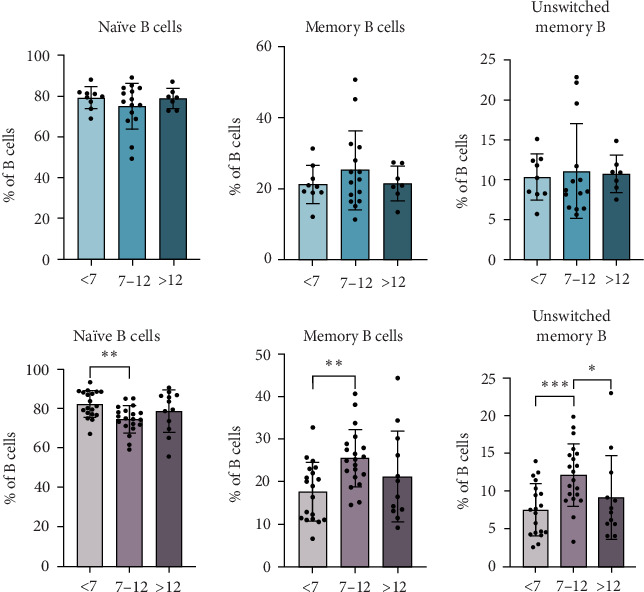
Age-related differences in memory and naïve B cells at diagnosis of T1D. The percentages of naïve and memory B cells, and within this group, of unswitched memory B cells, were determined in nondiabetic control subjects (blue bars) or at the time of T1D diagnosis (pink bars) and compared between age groups (<7, 7–12, and >12). *⁣*^*∗*^*p*  < 0.05, *⁣*^*∗∗*^*p*  < 0.01, *⁣*^*∗∗∗*^*p*  < 0.001, nonparametric *t*-test.

Other cells that significantly differed between age groups in patients with T1D were naïve and memory B cells (Figure 2). We found that patients between 7 and 12 years have significantly increased percentages of memory B cells, specifically the unswitched memory, when compared to the younger patient group. Respectively, they also showed decreased percentages of naïve B cells. Although these differences were not significant in the control subjects, a tendency for increased levels of memory B cells in the intermediate group can be noticed.

### 3.5. Myeloid Cells Remain Consistent Across Different Age Groups in T1D Patients

**Figure 3:**
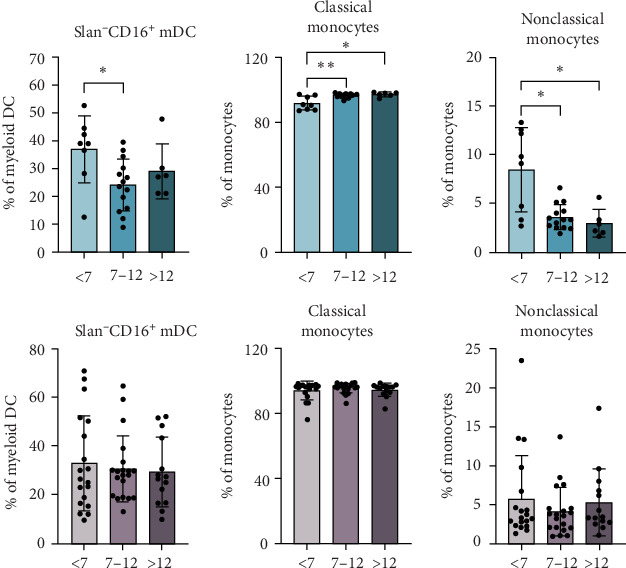
Age-related differences in myeloid DC and monocytes in nondiabetic controls. The percentages of Slan^−^CD16^+^ myeloid DCs and classical and nonclassical monocytes were determined in nondiabetic control subjects (blue bars) or at the time of T1D diagnosis (pink bars) and compared between age groups (<7, 7–12, and >12). *⁣*^*∗*^*p*  < 0.05, *⁣*^*∗∗*^*p*  < 0.01, nonparametric *t*-test.

Conversely, subpopulations of myeloid cells were also found to change in controls as they grow, but this does not occur in patients with T1D across the age groups. Although the lack of variation in these subpopulations does not help identify endotypes in patients with T1D, it does indicate a pathological process. In this sense, while the physiological process is to decrease both the percentages of Slan^−^CD16^+^ myeloid DC (mDC) and nonclassical monocytes and increase the percentage of classical monocytes with age, these differences are not noticed in patients with T1D (Figure 3).

### 3.6. The Percentage of NK Cells and Transitional B Cells Varies With Age in Both T1D Patients and Controls

**Figure 4:**
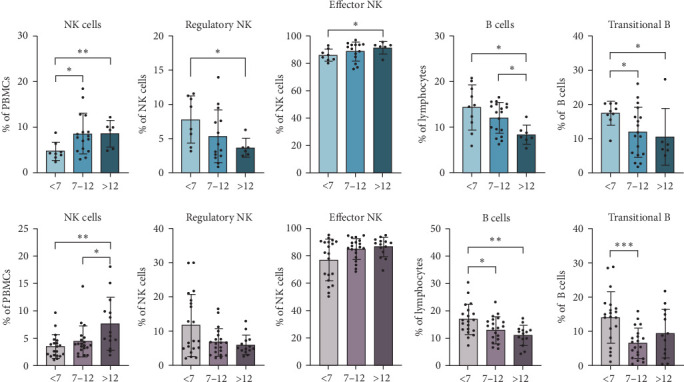
Shared age-related differences in B lymphocytes and NK cells between controls and patients with T1D. The percentages of total, regulatory, and effector NK cells and total and transitional B cells were determined in nondiabetic control subjects (blue bars) or at the time of T1D diagnosis (pink bars) and compared between age groups (<7, 7–12, and >12). *⁣*^*∗*^*p*  < 0.05, *⁣*^*∗∗*^*p*  < 0.01, *⁣*^*∗∗∗*^*p*  < 0.001, nonparametric *t*-test.

As expected, we also found lymphoid cells that vary with age in both patients with T1D and controls, indicating a physiological change rather than a distinct pathological process (Figure 4). In this sense, as the subject grows, the percentage of total NK cells increases. Within this subpopulation, regulatory NK cells decrease while effector NK cells increase. Additionally, as reported in various studies, total B lymphocytes decline with age, specifically the transitional naïve B lymphocytes.

### 3.7. The Frequency of Effector Memory (EM) CD4^+^ and CD8^+^ T Cells Tends to Increase With Age in Both T1D Patients and Controls

**Figure 5:**
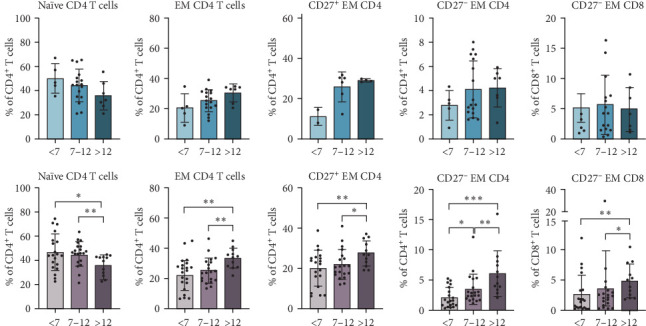
Shared age-related differences in CD4^+^ and CD8^+^ T lymphocytes between controls and patients with T1D. The percentages of naïve, EM (CD27^+^ and CD27^−^) CD + T cells and CD27^−^ EM CD8^+^ T cells were determined in nondiabetic control subjects (blue bars) or at the time of T1D diagnosis (pink bars) and compared between age groups (<7, 7–12, and >12). *⁣*^*∗*^*p*  < 0.05, *⁣*^*∗∗*^*p*  < 0.01, *⁣*^*∗∗∗*^*p*  < 0.001, nonparametric *t*-test.

Other subpopulations that change with age in patients with T1D and controls include EM CD4^+^ and CD8^+^ T cells, as well as their subtypes (whether or not they express CD27). Although we could not identify any statistically significant differences in the control group, the trend is evident and consistent with that observed in patients with T1D. In general, naïve CD4^+^ T cells decrease with age in both groups, whereas EM CD4^+^ T cells increase (both CD27^+^ and CD27^−^), finding statistically significant differences when comparing to the >12 group in patients with T1D (Figure 5). The same increase can be found for CD27^−^ EM CD8^+^ T cells, especially in patients >12 years in the T1D group.

## 4. Discussion

The development of T1D is influenced by factors such as age of onset, autoantibody profiles, immune regulation, β-cell destruction rates, genetics, and molecular mechanisms. Patients can be classified into endotypes based on these biological processes, which help explain the disease's complex pathophysiology. Age significantly contributes to T1D heterogeneity, with younger children showing faster progression, higher risk, and distinct genetic, immunological, and histological patterns [3]. Our findings support the existence of distinct endotypes of T1D related to age at diagnosis and define, for the first time, peripheral blood immune cell signatures in those children diagnosed before the age of 7, mainly characterized by decreased percentages of Tregs and Bregs.

Prior research has indicated the potential existence of age-related endotypes of T1D, with distinct etiopathological mechanisms observed in children diagnosed before the age of 7 compared to those diagnosed after 12 years [7]. Here, clinical features and metabolic data further underscore the age-related differences in T1D presentation and progression. Younger patients (<7 years) displayed more severe disease at diagnosis, as evidenced by shorter symptom duration, lower HCO_3_ and BE levels, and greater metabolic acidosis. Indeed, prior research has indicated that younger children are at an elevated risk for developing diabetic ketoacidosis at the time of diagnosis [23]. Additionally, they had lower C-peptide levels at diagnosis, indicating a more rapid and severe loss of β-cell function compared to older age groups. As other reports have shown, we did not find age-related differences regarding the frequency of familial T1D [24, 25]. HbA1c levels were also lower in younger patients at diagnosis, progressively increasing with age, as well as the duration of symptoms, which can reflect a faster transition to stage 3 and the need for insulin treatment.

1 year after diagnosis, metabolic control outcomes further highlighted age-related differences. C-peptide levels remained significantly lower in the <7 years group, indicating poorer β-cell preservation compared to older age groups, particularly patients >12 years. Glycemic control, as measured by HbA1c levels and TIR, was also superior in the >12 years group, suggesting that older patients achieve better metabolic stability. These findings suggest that age at diagnosis not only influences disease severity at onset but also impacts long-term β-cell function and glycemic outcomes. Moreover, age at diagnosis is a pivotal factor influencing the likelihood of remission. Typically, children exhibit a higher likelihood of experiencing the PR phase compared with adults, and within the former group, the probability of sustained remission tends to increase with higher onset age [26]. Here, we validated the fact that a diagnosis under the age of 7 is associated with a lower probability of presenting the PR phase, since their scarce pancreatic reserve is quickly exhaustible.

At the immunological level, autoantibodies have demonstrated age-specific incidence patterns that have been linked with particular rates of disease progression [27]. Our study identified increasing titers of IA2A at a younger age, in accordance with previous studies showing that patients diagnosed >12 years present lower titers of IA2A [28]. In fact, islet autoantibodies are lost over time in slow progressors [15]. Although we could not find differences in the frequency and titers of GAD65A, which in previous studies were found to be more prevalent in patients diagnosed >12 years and children with preclinical disease [29], the difference in the IA2A titers indicated heterogeneity in the initiation of the disease process.

Additionally, differences have been previously found in the level of pancreatic islet infiltration, with younger patients having a higher percentage of CD20^+^ B and CD8^+^ T lymphocytes and fewer insulin-containing islets [30]. However, little is known about the peripheral immunological changes that can reflect immune responses occurring in the patient in a noninvasive way. Here, we performed deep immunophenotyping on patients with T1D and control subjects to identify endotypes that simply reflect a pathobiological process, rather than changes in the immune system that are inherent to the patient's physiology and depend on their age. We found that patients <7 years exhibited reduced percentages of key regulatory immune cells, including memory and activated Tregs, Bregs, and Th17 cells, when compared to older age groups. This reduction in regulatory lymphocyte populations is consistent with the hypothesis that younger children may have a more profound immune dysregulation at disease onset. Given the pivotal function of Tregs and Bregs in maintaining immune tolerance, their diminished presence could contribute to the accelerated autoimmune destruction of pancreatic β-cells observed in early-onset T1D. Similarly, the observed decrease in Th17 cells in the youngest group may indicate an altered balance between pro-inflammatory and regulatory immune responses [31].

As expected, total B lymphocytes, particularly transitional B cells, decreased with age in both patients with T1D and controls. A tendency to decrease B cells in the blood with increasing age has already been described [32], which could also explain part of the higher proportion of B cells in the islets of the young. Therefore, we must be cautious when interpreting the reported findings of the insulitis profile. Nevertheless, patients aged 7–12 years exhibited significantly higher percentages of memory B cells, particularly unswitched memory B cells, and decreased percentages of naïve B cells compared to younger patients. Interestingly, this trend was not significant in age-matched control subjects. This finding indicates that older pediatric patients have a more mature B cell compartment, possibly influenced by prior antigenic exposure and immunological priming, and that the B cell compartment's maturation process may be accelerated or dysregulated in T1D, particularly in children diagnosed during the intermediate age group.

Total NK cells also increased with age in both T1D patients and healthy controls, accompanied by a reduction in regulatory NK cells and a rise in effector NK cells, confirming previously reported physiological patterns [33]. Moreover, both groups displayed age-related changes in EM CD4^+^ and CD8^+^ T cells, as previously reported for healthy children [32]. Naïve CD4^+^ T cells declined with age, while EM CD4^+^ and CD8^+^ T cells increased, with significant differences observed in the oldest group in T1D patients. These shared patterns highlight natural immune system maturation and indicate that the observed differences in other cell populations, such as Tregs, Bregs, and memory B cells, are likely specific to the pathogenesis of T1D rather than general developmental processes.

Finally, in contrast to lymphoid cells, myeloid cell populations exhibited no significant differences across the age groups in T1D patients. However, healthy controls demonstrated an age-related decline in Slan^−^CD16^+^ mDCs and nonclassical monocytes, alongside an increase in classical monocytes, which aligns with expected physiological maturation [32]. The absence of these changes in T1D patients suggests a potential disruption in the natural progression of myeloid cell development, irrespective of age, further supporting the notion of an underlying pathological process associated with T1D. In fact, there is a heightened monocyte proinflammatory/cytolytic activity with T1D susceptibility and progression [34]. While these findings do not delineate distinct age-related endotypes, they point toward broader immune dysregulation in pediatric T1D.

In conclusion, our work in defining age-related peripheral immune endotypes improves our understanding of disease progression and enables more accurate monitoring and patient-centered therapies. Our findings support the hypothesis that children diagnosed before the age of seven exhibit a more aggressive form of the disease, characterized by reduced regulatory T and B lymphocytes, Th17 and memory B cells, higher IA2A titers, lower pancreatic reserve, and rapid exhaustion within 1 year. These children require prompt, efficient treatment due to their unfavorable prognosis. Additionally, noninvasive techniques can identify these endotypes, which reflect distinct immunopathological processes. This highlights the need to distinguish between physiological changes and disease-specific immune alterations in future trials.

## Figures and Tables

**Table 1:** Sociodemographic, clinical, metabolic, and immunological data at T1D diagnosis in patients at baseline and divided by age groups.

Parameter	Baseline T1D (*n* = 67)	<7 years (*n* = 21)	7–12 years (*n* = 26)	>12 years (*n* = 20)	*p*-Value (<7 vs. 7–12)	*p*-Value (<7 vs. >12)	*p*-Value (7–12 vs. >12)
Age (years)	9.7 (6.4–13.4)	4.3 (2.4–6.4)	9.8 (8.3–11.8)	15.2 (13.8–16.9)	**<0.0001**	**<0.0001**	**<0.0001**
BMI SDS	−0.5 (−1.1 to −0.1)	−0.3 (−0.94 to −0.29)	−0.52 (−1.2 to −0.08)	−0.52 (−1.1 to −0.04)	0.6832	0.4773	0.8564
# Male sex (%)	38 (56.7%)	15 (71.4%)	11 (42.3%)	12 (60%)	0.0762	0.5204	0.3726
Relatives with T1D (%)	6 (8.96%)	1 (4.7%)	4 (15%)	1 (5%)	0.3623	1.0000	0.3693
Celiac and/or tiroiditis (%)	7 (10,4%)	1 (4.8%)	5 (19.2%)	1 (5%)	0.2044	1.0000	0.2122
Time previous symptoms (weeks)	4.6 (2–4)	2.2 (1–3)	5.3 (2–4)	6.4 (2–11)	**0.0331**	**0.0397**	0.8258
HCO_3_ (mmol/L)	17.6 (12.4–23.5)	13.9 (6.2–21.3)	18.2 (13.1–22.2)	20.9 (17.2–26.6)	0.0839	**0.0034**	0.0849
BE	−8.66 (−13.5 to −2)	−13.1 (−21.7 to −2.5)	−7.41 (−12.3 to −2.4)	−5.6 (−8.7 to −0.2)	0.1031	**0.** **0067**	0.0770
Insulin dose	0.68 (0.53–0.83)	0.65 (0.55–0.8)	0.69 (0.5–0.8)	0.706 (0.53–0.83)	0.7544	0.5829	0.8909
C-peptide (ng/mL)	0.4 (0.2–0.5)	0.27 (0.1–0.3)	0.39 (0.2–0.5)	0.56 (0.3–0.6)	**0.0341**	**0.0002**	0.0795
HbA1c (%)	11.5 (10.1–13.2)	10.3 (9.4–10.6)	11.9 (10.7–13.1)	12.4 (10.7–14.3)	**0.00** **01**	**0** **.0030**	0.3641
IA2A positives (%)	46 (68.7%)	16 (76.2%)	20 (76.9%)	10 (50%)	1.0000	0.1109	0.07013
IA2A (U/mL)	77.2 (0.7–127.4)	102.6 (2–179)	87.2 (1.27–129)	42.7 (0.75–56)	0.9113	0.0814	**0.0209**
GAD65A positives (%)	57 (85.1%)	18 (85.7%)	23 (88.5%)	16 (80%)	1.0000	0.6965	0.6816
GAD65A (U/mL)	51 (10.5–107)	42.1 (8.9–54.2)	61.1 (13.9–107.6)	47.6 (7.4–84.4)	0.6131	0.8613	0.4334

*Note:* Data are presented as mean and interquartile ranges (Q1–Q3) for nonparametric variables. *p*-Value from Mann–Whitney *U* test for continous variables and from Fisher's exact test for categorical variables. HbA1c, glycated hemoglobin; HCO_3_, bicarbonate. Bold indicates statistically significant values.

Abbreviations: BE, base excess; BMI, body mass index.

**Table 2:** Metabolic data 12 months after the diagnosis of T1D in patients divided by age groups.

Parameter	<7 years at 12 months (*n* = 21)	7–12 years at 12 months (*n* = 25)	>12 years at 12 months (*n* = 20)	*p*-Value (<7 vs. 7–12)	*p*-Value (<7 vs. >12)	*p*-Value (7–12 vs. >12)
C-peptide (ng/mL)	0.1 (0.01–0.1)	0.46 (0.2–0.65)	0.95 (0.4–1.5)	**<0.0001**	**<0.0001**	**0.0253**
HbA1c (%)	7.6 (7–8.1)	7.6 (7.1–8.5)	6.7 (5.9–7.2)	0.8741	**0.0021**	**0.0008**
% TIR	51.7 (41.5–63)	47.8 (30–60)	67.4 (53–75)	0.5610	**0.0136**	**0.00** **51**

*Note:* Data are presented as median and interquartile ranges for nonparametric variables. *p*-Value from Mann–Whitney *U* test. HbA1c, glycated hemoglobin. Bold indicates statistically significant values.

Abbreviation: TIR, time in range.

**Table 3:** Partial remission frequencies in patients with T1D divided by age groups at diagnosis.

Parameter	<7 (*n* = 21)	7–12 (*n* = 26)	>12 (*n* = 20)	*p*-Value (<7 vs. >12)
Remission	11 (52.4%)	17 (65.4%)	17 (85%)	**0.0431**
Nonremission	10 (47.6%)	9 (34.6%)	3 (15%)	

*Note:* Data presented as total number and percentage (%). *p*-Value from Fisher's exact test. Bold indicates statistically significant values.

## Data Availability

The data underlying the findings of this study are provided within the article. For further information or questions regarding the data, please contact the corresponding author.

## Nomenclature

BE: Base excess
BMI: Body mass index
Bregs: Regulatory B lymphocytes
DC: Dendritic cells
EM: Effector memory
GAD65A: Glutamic acid decarboxylase 65 autoantibodies
HbA1c: Glycated hemoglobin
IA2A: Insulinoma antigen 2 autoantibodies
IAAs: Insulin autoantibodies
IDAA1c: Insulin-dose adjusted glycated hemoglobin
mDC: Myeloid dendritic cells
NK: Natural killer
PR: Partial remission
SDS: SD score
T1D: Type 1 diabetes
T1DE1: Type 1 diabetes endotype 1
T1DE2: Type 1 diabetes endotype 1
TEDDY: The environmental determinants of diabetes in the young
TIR: Time in range
Tregs: Regulatory T lymphocytes.
